# Effects of Equol on Hand Osteoarthritis in Perimenopausal Women: A Pilot Study

**DOI:** 10.7759/cureus.88013

**Published:** 2025-07-15

**Authors:** Takashi Shimoe, Hiroshi Hashizume, Hiroyuki Oka, Tadafumi Susa, Tomomi Ueno, Shigeto Uchiyama, Yasushi Toge, Mito Hayashi, Yoji Kitano, Hiroshi Yamada

**Affiliations:** 1 Department of Orthopaedic Surgery, Wakayama Medical University, Wakayama, JPN; 2 Division of Musculoskeletal AI System Development, Faculty of Medicine, The University of Tokyo, Tokyo, JPN; 3 Department of Orthopaedic Surgery, Susa Hospital, Wakayama, JPN; 4 Saga Nutraceuticals Research Institute, Otsuka Pharmaceutical Co. Ltd., Saga, JPN; 5 Department of Orthopaedic Surgery, Wakayama Rosai Hospital, Wakayama, JPN; 6 Department of Orthopaedic Surgery, Hashimoto Municipal Hospital, Wakayama, JPN; 7 Department of Orthopaedic Surgery, Shingu Municipal Medical Center, Wakayama, JPN

**Keywords:** equol, hand osteoarthritis, menopause, pain, women

## Abstract

Background: Hand pain in women has recently been reported as a symptom of menopause, and the sudden decrease in estrogen levels may be responsible for joint pain in the hand. We therefore hypothesized that intake of the estrogen analog equol (4',7-isoflavandiol) would improve pain resulting from osteoarthritis of the hand in menopausal women. We evaluate the efficacy of equol intake in improving pain and disabilities of daily living caused by hand osteoarthritis in perimenopausal women.

Methods: Women aged 45-60 years with hand pain were recruited for provisional registration (n=148). Hand osteoarthritis was diagnosed with radiography according to the Kellgren-Lawrence grading system. Excluded from the study were patients whose laboratory data indicated they had rheumatoid arthritis, thyroid disorders, or naturally possessed equol. Finally, 104 participants were registered for the protocol therapy, and we recorded visual analogue scale (0-100 mm) data on the movement and Disabilities of the Arm, Shoulder, and Hand (DASH) score after 12 weeks of equol intake versus baseline.

Results: Change in visual analogue scale on movement after 12 weeks of equol intake was -34.0 mm relative to baseline (*p*<0.001) and disability of arm, shoulder, and hands score after 12 weeks of equol intake compared with baseline was -6.6 (*p*<0.001).

Conclusions: This is the first prospective clinical trial of an equol supplement as a treatment for pain caused by hand osteoarthritis. Visual analogue scale on movement improved with a statistically significant difference. The improvement was observed within 1-3 months after intake of equol. The DASH score also showed significant improvement. Equol is suggested to improve hand pain, upper limb function, and quality of life.

## Introduction

Hand osteoarthritis (HOA) localized to the distal interphalangeal (DIP), proximal interphalangeal (PIP), and metacarpophalangeal (MCP) joints, as well as the carpometacarpal (CMC) joint of the thumb, has been treated with analgesics and other drug therapies, and with non-pharmacologic treatment such as splints. Some patients respond well to medication and orthotics, but in general, the effectiveness of these therapies is limited. Surgical treatment, such as arthroplasty or arthrodesis, is a consideration, but is indicated for/requested by few patients due to the long-term limitation of hand use in the perioperative period and because of the potential development of limited range of motion and decreased dexterity [1].

Hand pain in women has recently been reported as a symptom of menopause; the sudden decrease in estrogen levels may be responsible for joint pain in the hand [2]. Estrogen deficiency is thought to affect specific hand joints, particularly the DIP, PIP joints, and the CMC joint of the thumb, which are commonly involved in HOA. Epidemiological studies have also suggested that the prevalence and severity of osteoarthritis in general may increase after menopause due to decreased estrogen levels, which can affect cartilage metabolism and joint structure [3]. Some evidence indicates that hormone replacement therapy (HRT) may reduce the risk or progression of OA, although findings remain inconclusive [3]. Estrogen receptors include α-receptors and β-receptors: α-receptors are mainly distributed in organs such as the ovaries and the mammary glands, while β-receptors are distributed in tissues such as bone and synovial membrane [4].

We therefore hypothesized that intake of the estrogen analog equol (4',7-isoflavandiol) would improve pain resulting from HOA in menopausal women. Equol is a substance produced when daidzein, a type of soy isoflavone, is metabolized by intestinal bacteria. Some people naturally possess intestinal bacteria that metabolize equol from daidzein (equol producers), and others do not (equol non-producers) [5].

Equol has been reported to alleviate menopausal symptoms such as hot flashes and neck stiffness [[6]]. The marketed S-equol selectively binds to the β-receptor with high affinity [7-9]. Equol is expected to improve hand symptoms in perimenopausal women because it acts mainly on bone and synovium, where β-receptors are present, and there is little effect on the reproductive system.

This study aims to evaluate the efficacy of equol intake in improving pain and disabilities of daily living caused by HOA in perimenopausal women. It is the first prospective clinical trial of equol supplement as a treatment for pain caused by HOA. This study was conducted as a pilot study as preparation for a future randomized controlled trial. The primary objective of this pilot study was to investigate the potential efficacy of equol supplementation in improving pain and upper limb function in perimenopausal women with HOA.

This article was previously presented as a meeting abstract at the Federation of European Societies for the Surgery of the Hand (FESSH) 2024 Congress on June 28, 2024.

## Materials and methods

Patients and design

A multicenter single-arm phase II trial was designed to evaluate the efficacy of equol intake for HOA in perimenopausal women. The study population consisted of Japanese perimenopausal women aged 45-60 years, with no prior history of hand surgery or inflammatory joint disease. All participants were screened by clinical and radiographic assessment to confirm the diagnosis of HOA. Radiographic assessment was performed by determining the Kellgren-Lawrence (KL) grade [10] for 30 joints in both hands, including the thumb IP, MP, and CMC joints as well as the DIP, PIP, and MP joints of the index to little fingers. The KL classification was used to assess the severity of osteoarthritis, with grade 0 indicating no radiographic features, grade 1 doubtful narrowing of joint space and possible osteophytic lipping, grade 2 definite osteophytes and possible joint space narrowing, grade 3 multiple moderate osteophytes, definite joint space narrowing, some sclerosis, and possible bony deformity, and grade 4 large osteophytes, marked joint space narrowing, severe sclerosis, and definite bony deformity. Radiographic assessment using the KL grading system was performed by the corresponding author, who has extensive experience in diagnosing hand osteoarthritis. The readings were not blinded in this pilot study.

Provisionally enrolled in the study were 148 Japanese women aged 45 to 60 years with hand pain localized to the distal interphalangeal (DIP), proximal interphalangeal (PIP), and metacarpophalangeal (MCP) joints, as well as the carpometacarpal (CMC) joint of the thumb. Pain was defined as persistent discomfort or aching during daily movements and assessed using a visual analog scale (VAS). Only patients with pain lasting more than 3 months were considered eligible. They were examined by urinalysis for the ability to produce equol and by blood test for the presence of findings suggestive of thyroid dysfunction, rheumatoid arthritis, or other collagen diseases. Among them, 104 patients who were non-producers of equol and were not suspected of having a history of rheumatoid arthritis or other collagen diseases were enrolled and started the protocol treatment. Data were collected between November 2019 and September 2021. The research was approved by the certified review board for the specified clinical trials or ethics committee, and was performed in compliance with the 1964 Declaration of Helsinki and its later amendments. Each patient provided written informed consent. Inclusion criteria were women aged 45-60 years with hand joint pain lasting more than three months, a KL grade of 0 or higher, non-producers of equol, and availability for three months of follow-up.

Exclusion criteria for this research were surgical menopause, rheumatoid arthritis or other collagen diseases, thyroid dysfunction, history of hand trauma as the cause of current pain or deformity of the hands, mental problems more than 11 points on Hospital Anxiety and Depression Scale [11], allergy to soy, use of prescriptions for menopausal symptoms (including hormonal agents, herbal medicine and SERMs), use of food products containing equol other than the test supplement (trade name: Equelle, Otsuka Pharmaceutical Co., Ltd, Tokyo, Japan), chronic use of analgesics, current use of oral contraceptives, pregnancy or lactating, and patients who are otherwise judged by the researcher to be inappropriate for inclusion in this study. Patients with thyroid dysfunction were excluded from this study because it has been shown that some patients with HOA may develop it from a background of hypothyroidism, and there may be a different etiology from generalized HOA [12-13].

The patients' ability to produce equol was assessed by urinalysis. After obtaining consent to participation in the study, we examined the capacity to produce equol by testing urinary equol and isoflavone in the first morning urine on the day following consumption of sufficient soy foods (at least one of the following: 150 g tofu, 40 g nattō, or 200 mL soy milk) at lunch or dinner. Equol production capacity was determined by the log ratio of equol concentration (nmol/mL) to daidzein concentration (nmol/mL) using the concentration of daidzein (a type of soy isoflavone), which is a substrate of equol, according to a previously-reported method [14] Equol-producers were defined as those with a log₁₀-transformed ratio of equol (nmol/mL) to daidzein (nmol/mL) of -1.75 or higher, and non-producers as those with a ratio below -1.75. This assessment confirmed that participants were equol non-producers. Equol producers can naturally produce equol through dietary soy intake, which could affect blood equol levels and confound the study results. Therefore, by administering equol tablets to non-producers only, we ensured a consistent and controlled intake level to accurately evaluate its effects.

Women aged 45-60 years were included in this study because, in a 2012 report, the median age of menopause for Japanese women was 52.1 years, with the 25th percentile menopausal age reported as 50.3 years and the 75th percentile menopausal age as 53.8 years [15]. The menopausal period is defined as the period including five years before and five years after menopause itself (the final menstruation), and thus the perimenopausal phase can be considered to affect women aged between 45 and 60.

The Hospital Anxiety and Depression Scale consists of 14 multiple-choice questions divided into two subscales, one of anxiety and one of depression, with seven questions in each. The total score of each subscale ranges from 0 to 21 points, and it is used to detect mild degrees of affective disorders in a non-psychiatric environment. With the criteria described to them, women aged 45-60 years with pain in their hands were recruited for provisional registration (n=148).

**Figure 1:**
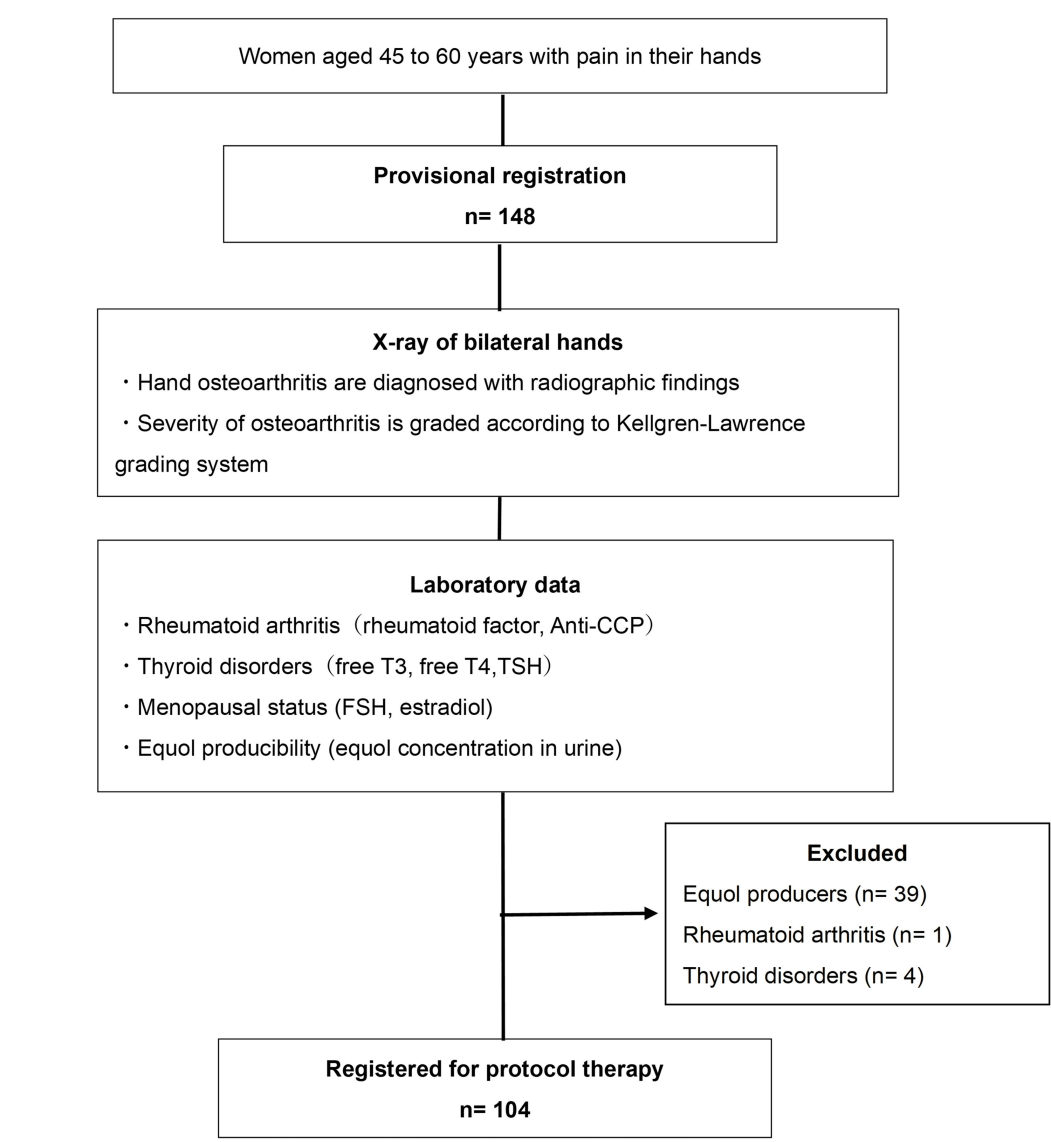
Patient flow diagram.

HOA was diagnosed with radiography according to the KL grading system. Laboratory data related to rheumatoid arthritis, thyroid disorders, and equol producibility were investigated. We excluded patients who had rheumatoid arthritis (n=1), thyroid disorders (n=4), and those who naturally produced equol (n=39). Finally, 104 participants were registered for protocol therapy (Figure 1).

This study was conducted as a single-arm, open-label pilot trial; therefore, no randomization or blinding procedures were applied. Participants who did not complete the 12-week intervention were excluded from the final analysis, and all analyses were conducted on a per-protocol basis.

Protocol therapy

Participants confirmed as equol non-producers took equol 10 mg (2.5 mg × four tablets) orally every morning and evening with meals, taking two tablets each time, for a total of 12 weeks. Participants were instructed to record missed doses and were allowed to take the missed tablets later the same day, not exceeding four tablets per day. Compliance was monitored by tablet count and participant diaries. The dosage regimen was the same as in the previously reported study for improvement of general menopausal symptoms: two tablets, twice a day (after breakfast and dinner). The intake period was also set to 12 weeks, as in the previous study [6]. Two of the 104 patients who registered for protocol therapy were unable to complete the protocol treatment and were excluded from the statistical analysis (Table 1, 2).

**Table 1:** The number of patients for each analysis.

Type of analysis	n
Full analysis set	104
Safety analysis set	102
Per protocol set	102

**Table 2:** Baseline patient characteristics in the per-protocol analysis set. SD= standard deviation

Variables	Per protocol set
	n= 102
Sex (female), %	100
Age, mean ± SD	53.6 ± 4.27

Measurements

The primary endpoint was the change in VAS on movement after 12 weeks of equol intake relative to the baseline VAS on movement.

Secondary endpoints were the change in VAS on movement and at rest at 4, 8, and 12 weeks post-equol intake relative to baseline, the number of painful bilateral hand joints, which included the thumb IP, MP, and CMC joints, and the DIP, PIP, and MP joints of the index to little fingers (for a total of 15 joints per hand and 30 joints bilaterally), an evaluation index of quality of life (EQ-5D-5L health states in Japan [16]) and the upper extremity disability assessment chart (disability of the arm, shoulder and hand questionnaire; also known as ‘DASH’, Japanese Society for Surgery of the Hand version) [17] at 4, 8, and 12 weeks post-equol intake compared with the baseline. We evaluated the correlation between VAS changes on movement after 12 weeks of equol intake and maximum KL grade at baseline, and also the sum of KL grades (total KL score) of the 30 joints of both hands. In this study, we investigated not only hand pain but also other systemic menopausal symptoms. The simplified menopausal index (SMI) scoring 10 menopausal symptoms (hot flashes, sweating, insomnia, headache, dizziness, palpitation, joint pain, fatigue, irritability, and depression) [18], and each major menopausal symptom was assessed by VAS. Patient satisfaction with treatment was rated on a 5-point scale from 1 (satisfied) to 5 (dissatisfied) after 12 weeks of taking equol. Safety endpoints included adverse events requiring medical treatment that occurred during the protocol therapy with equol for 12 weeks or by the time of discontinuation of the protocol therapy.

Statistical analysis

For the primary endpoint, summary statistics (mean, standard deviation, minimum, median, and maximum), 95% confidence intervals, and paired t-test p-values were calculated for the cases with at least 90% intake of the test food (n=97) (Table 3).

**Table 3:** Intake rate of test food for safety analysis set. SD= standard deviation

Intake rate of test food	n= 102
mean ± SD	98.2 ± 3.10
<90％ (%)	5 (4.9)
90％≦ (%)	97 (95.1)

Summary statistics were calculated at each time point for each of the secondary endpoints (VAS on movement, VAS at rest, number of painful finger joints, EQ-5D-5L, DASH score, simplified menopausal index, menopausal symptoms VAS, and treatment satisfaction) for the cases including intake of the test food <90% (n= 102). Scatter plots of VAS change on movement and items at baseline (maximum KL grade, total KL score, and treatment satisfaction) were generated after 12 weeks of equol intake.

## Results

As the primary endpoint, the change in VAS on movement after 12 weeks of equol intake relative to the VAS on movement in baseline was -34.0 ± 23.5 mm (Table 4).

**Table 4:** Primary endpoint. Change in visual analog scale on movement. VAS= visual analog scale; SD= standard deviation; mean (95%CI)=mean value with 95% confidence interval;  minimum/median/maximum= observed range and median of the VAS score; p-value= paired t-test comparing screening and 12 weeks. Outcome = screening and 12-week measured values, variation, and percentage of subjects showing an improvement of ≥20 mm in VAS.

Intake rate of test food	Outcome	n	VAS mean (95%CI; mm)	p-value	VAS SD (mm)	VAS minimum (mm)	VAS Median (mm)	VAS maximum (mm)
90％≦	Measured value (screening)	97	62.8 (58.96, 66.59)	-	19.19	9	64	100
	Measured value (12 weeks)	97	28.8 (23.72, 33.91)	-	25.6	0	21	95
	Variation	97	-34.0 (-38.63, -29.28)	<0.0001	23.49	-85	-32	18
	Variation > 20 (%)	73 (75.3)						

The change in VAS at rest after 12 weeks of equol intake relative to the VAS at rest in baseline was -23.5 ± 26.8 mm. The change in DASH score after 12 weeks of equol intake relative to the DASH score at baseline was -6.6 ± 10.7. Of the VAS items related to menopause, only depression was shown to have no statistically significant difference after 12 weeks compared with the baseline; all other secondary endpoints showed statistically significant improvement (p<0.05). For each endpoint, there was improvement over time from the start of equal tablet intake at 4, 8, and 12 weeks (Table 5).

**Table 5:** Secondary endpoints. VAS= visual analog scale; SD= standard deviation; mean (95%CI)= mean value with 95% confidence interval;  minimum/median/maximum= observed range and median of the VAS score; p-value= paired t-test comparing screening and 12 weeks; DASH= disability of the arm, shoulder and hands; SMI= simplified menopausal index (range: 0-100); DIP= distal; interphalangeal; PIP= proximal interpharangeal; CMC= carpometacarpal. *The SMI consists of 10 common menopausal symptoms widely used in Japan (see Koyama [18]). **Treatment satisfaction was rated on a 5-point scale: 1 = Satisfied with treatment, no complaints at all; 2 = Generally satisfied; 3 = Neither satisfied nor dissatisfied; 4 = Somewhat satisfied but dissatisfied; 5 = Not satisfied.

Variables	At the point		n	VAS mean [95%CI] (mm)	p-value	VAS SD (mm)	VAS minimum (mm)	VAS median (mm)	VAS maximum (mm)
VAS on movement	Screening	Measured value	102	62.6 (58.95, 66.26)	-	18.84	9	63.5	100
	4 weeks	Measured value	102	47.4 (42.64, 52.18)	-	24.57	0	50	100
		Variation	102	-15.2 (-18.76, -11.63)	<0.0001	18.35	-68	-14	19
	8 weeks	Measured value	102	36.7 (31.54, 41.83)	-	26.52	0	31	100
		Variation	102	-25.9 (-30.28, -21.56)	<0.0001	22.45	-78	-25	28
	12 weeks	Measured value	102	30.0 (25.01, 35.05)	-	25.88	0	25	95
		Variation	102	-32.6 (-37.27, -27.89)	<0.0001	24.15	-85	-32	18
VAS at rest	Screening	Measured value	102	36.8 (31.38, 42.15)	-	27.76	0	30.5	100
	4 weeks	Measured value	102	25.3 (20.49, 30.10)	-	24.78	0	21	94
		Variation	102	-11.5 (-15.63, -7.31)	<0.0001	21.43	-79	-10	67
	8 weeks	Measured value	102	15.8 (11.88, 19.65)	-	20.01	0	6	85
		Variation	102	-21.0 (-25.78, -16.22)	<0.0001	24.61	-81	-15	57
	12 weeks	Measured value	102	13.3 (9.43, 17.08)	-	19.69	0	3.5	93
		Variation	102	-23.5 (-28.70, -18.32)	<0.0001	26.75	-92	-19	50
EQ-5D-5L	Screening	Measured value	102	0.828 (0.8112, 0.8456)	-	0.0885	0.48	0.8704	0.938
	4 weeks	Measured value	102	0.860 (0.8460, 0.8738)	-	0.0715	0.587	0.8978	0.938
		Variation	102	0.031 (0.0176, 0.0454)	<0.0001	0.0714	-0.116	0.0074	0.323
	8 weeks	Measured value	102	0.861 (0.8460, 0.8765)	-	0.0786	0.574	0.8978	0.938
		Variation	102	0.033 (0.0177, 0.0481)	<0.0001	0.0783	-0.182	0.0274	0.418
	12 weeks	Measured value	102	0.867 (0.8519, 0.8829)	-	0.0798	0.455	0.8978	0.938
		Variation	102	0.039 (0.0213, 0.0568)	<0.0001	0.0914	-0.268	0.0274	0.418
DASH	Screening	Measured value	101	17.1 (14.55, 19.56)	-	12.85	1.7	14.17	62.5
	4 weeks	Measured value	102	13.5 (11.30, 15.63)	-	11.17	0	11.21	65.8
		Variation	101	-3.6 (-4.96, -2.17)	<0.0001	7.14	-41.7	-3.33	11.8
	8 weeks	Measured value	102	11.3 (9.11, 13.55)	-	11.42	0	8.18	65.5
		Variation	101	-5.7 (-7.61, -3.76)	<0.0001	9.88	-55	-4.17	23.9
	12 weeks	Measured value	102	10.4 (8.32, 12.56)	-	10.92	0	7.2	56.9
		Variation	101	-6.6 (-8.68, -4.52)	<0.0001	10.66	-57.5	-4.83	35.8
SMI*	Screening	Measured value	102	28.0 (24.47, 31.45)	-	17.98	3	24	92
	4 weeks	Measured value	102	22.7 (19.53, 25.89)	-	16.39	3	18	81
		Variation	102	-5.3 (-6.94, -3.57)	<0.0001	8.67	-33	-3	18
	8 weeks	Measured value	102	21.7 (18.59, 24.74)	-	15.85	3	18	88
		Variation	102	-6.3 (-8.22, -4.37)	<0.0001	9.92	-41	-6	16
	12 weeks	Measured value	102	20.2 (17.07, 23.30)	-	16.06	3	15	86
		Variation	102	-7.8 (-10.14, -5.41)	<0.0001	12.19	-49	-6	31
VAS for hot flushes	Screening	Measured value	102	18.9 (14.21, 23.61)	-	24.21	0	9	90
	4 weeks	Measured value	102	16.0 (11.71, 20.31)	-	22.18	0	6.5	100
		Variation	102	-2.9 (-6.59, 0.79)	0.1266	19.03	-62	0	100
	8 weeks	Measured value	102	12.3 (8.66, 15.97)	-	18.84	0	4	77
		Variation	102	-6.6 (-10.39, -2.80)	0.0009	19.56	-68	0	65
	12 weeks	Measured value	102	11.6 (7.75, 15.48)	-	19.91	0	2	100
		Variation	102	-7.3 (-11.64, -2.95)	0.0014	22.39	-75	-1	100
VAS for abnormal sweating	Screening	Measured value	102	23.6 (18.39, 28.83)	-	26.91	0	15.5	91
	4 weeks	Measured value	102	18.3 (13.71, 22.94)	-	23.79	0	8	100
		Variation	102	-5.3 (-9.59, -0.98)	0.0179	22.18	-75	0	100
	8 weeks	Measured value	102	18.4 (13.66, 23.12)	-	24.36	0	6.5	100
		Variation	102	-5.2 (-10.41, -0.02)	0.0517	26.76	-80	0	82
	12 weeks	Measured value	102	15.3 (10.80, 19.77)	-	23.13	0	5	100
		Variation	102	-8.3 (-13.40, -3.25)	0.0018	26.15	-85	-1	91
VAS for insomnia	Screening	Measured value	102	24.0 (18.27, 29.69)	-	29.4	0	9	100
	4 weeks	Measured value	102	20.2 (14.71, 25.61)	-	28.09	0	4	100
		Variation	102	-3.8 (-7.33, -0.32)	0.0349	18.06	-59	0	77
	8 weeks	Measured value	102	17.5 (12.51, 22.55)	-	25.85	0	1	93
		Variation	102	-6.5 (-10.35, -2.55)	0.0016	20.09	-69	0	46
	12 weeks	Measured value	102	17.3 (11.99, 22.56)	-	27.24	0	2	95
		Variation	102	-6.7 (-11.11, -2.30)	0.0036	22.7	-75	0	82
VAS for melancholy	Screening	Measured value	102	5.4 (3.03, 7.76)	-	12.18	0	0	79
	4 weeks	Measured value	102	6.5 (3.54, 9.48)	-	15.29	0	0	100
		Variation	102	1.1 (-1.87, 4.10)	0.4650	15.39	-53	0	100
	8 weeks	Measured value	102	3.9 (2.05, 5.77)	-	9.59	0	0	52
		Variation	102	-1.5 (-3.82, 0.86)	0.2171	12.04	-72	0	31
	12 weeks	Measured value	102	7.0 (3.50, 10.42)	-	17.82	0	0	100
		Variation	102	1.6 (-2.03, 5.17)	0.3954	18.56	-66	0	100
VAS for stiff shoulders and neck	Screening	Measured value	102	48.3 (42.74, 53.86)	-	28.65	0	51.5	100
	4 weeks	Measured value	102	40.0 (34.44, 45.62)	-	28.8	0	33.5	100
		Variation	102	-8.3 (-11.93, -4.62)	<0.0001	18.83	-58	-5.5	70
	8 weeks	Measured value	102	35.9 (30.34, 41.43)	-	28.57	0	30.5	100
		Variation	102	-12.4 (-16.72, -8.12)	<0.0001	22.14	-76	-8.5	51
	12 weeks	Measured value	102	34.3 (28.49, 40.14)	-	30.01	0	25.5	100
		Variation	102	-14.0 (-18.94, -9.04)	<0.0001	25.5	-91	-11.5	66
Treatment safisfaction**	12 weeks	Measured value	102	1.5 (1.37, 1.70)	-	0.85	1	1	4
Total number of painful joints	Screening	Measured value	102	6.8 (5.77, 7.90)	-	5.5	1	5	30
	4 weeks	Measured value	102	5.4 (4.47, 6.27)	-	4.63	0	4	24
		Variation	102	-1.5 (-1.95, -0.97)	<0.0001	2.54	-11	-1	3
	8 weeks	Measured value	102	4.9 (4.01, 5.74)	-	4.47	0	3	24
		Variation	102	-2.0 (-2.55, -1.37)	<0.0001	3.02	-12	-1	3
	12 weeks	Measured value	102	4.4 (3.54, 5.18)	-	4.22	0	3	24
		Variation	102	-2.5 (-3.14, -1.80)	<0.0001	3.47	-16	-2	5
Number of painful DIP joints	Screening	Measured value	102	4.1 (3.53, 4.75)	-	3.13	0	4	10
	4 weeks	Measured value	102	3.2 (2.67, 3.76)	-	2.79	0	2.5	10
		Variation	102	-0.9 (-1.30, -0.55)	<0.0001	1.93	-7	0	4
	8 weeks	Measured value	102	2.9 (2.41, 3.42)	-	2.6	0	2	10
		Variation	102	-1.2 (-1.64, -0.81)	<0.0001	2.13	-8	-1	3
	12 weeks	Measured value	102	2.6 (2.06, 3.06)	-	2.57	0	2	10
		Variation	102	-1.6 (-2.02, -1.14)	<0.0001	2.27	-9	-1	5
Number of painful PIP joints	Screening	Measured value	102	1.4 (0.93, 1.85)	-	2.38	0	0	8
	4 weeks	Measured value	102	1.1 (0.73, 1.56)	-	2.13	0	0	8
		Variation	102	-0.2 (-0.41, -0.08)	0.0055	0.87	-3	0	4
	8 weeks	Measured value	102	1.1 (0.70, 1.46)	-	1.95	0	0	8
		Variation	102	-0.3 (-0.55, -0.08)	0.0097	1.2	-7	0	3
	12 weeks	Measured value	102	0.9 (0.58, 1.30)	-	1.85	0	0	8
		Variation	102	-0.5 (-0.72, -0.18)	0.0014	1.38	-7	0	3
Number of painful CMC joints of thumb	Screening	Measured value	102	0.5 (0.39, 0.69)	-	0.78	0	0	2
	4 weeks	Measured value	102	0.5 (0.34, 0.64)	-	0.77	0	0	2
		Variation	102	0.0 (-0.16, 0.06)	0.4007	0.59	-2	0	2
	8 weeks	Measured value	102	0.4 (0.26, 0.52)	-	0.66	0	0	2
		Variation	102	-0.1 (-0.27, -0.02)	0.0214	0.64	-2	0	2
	12 weeks	Measured value	102	0.4 (0.23, 0.48)	-	0.65	0	0	2
		Variation	102	-0.2 (-0.30, -0.07)	0.0020	0.59	-2	0	1

**Figure 2:**
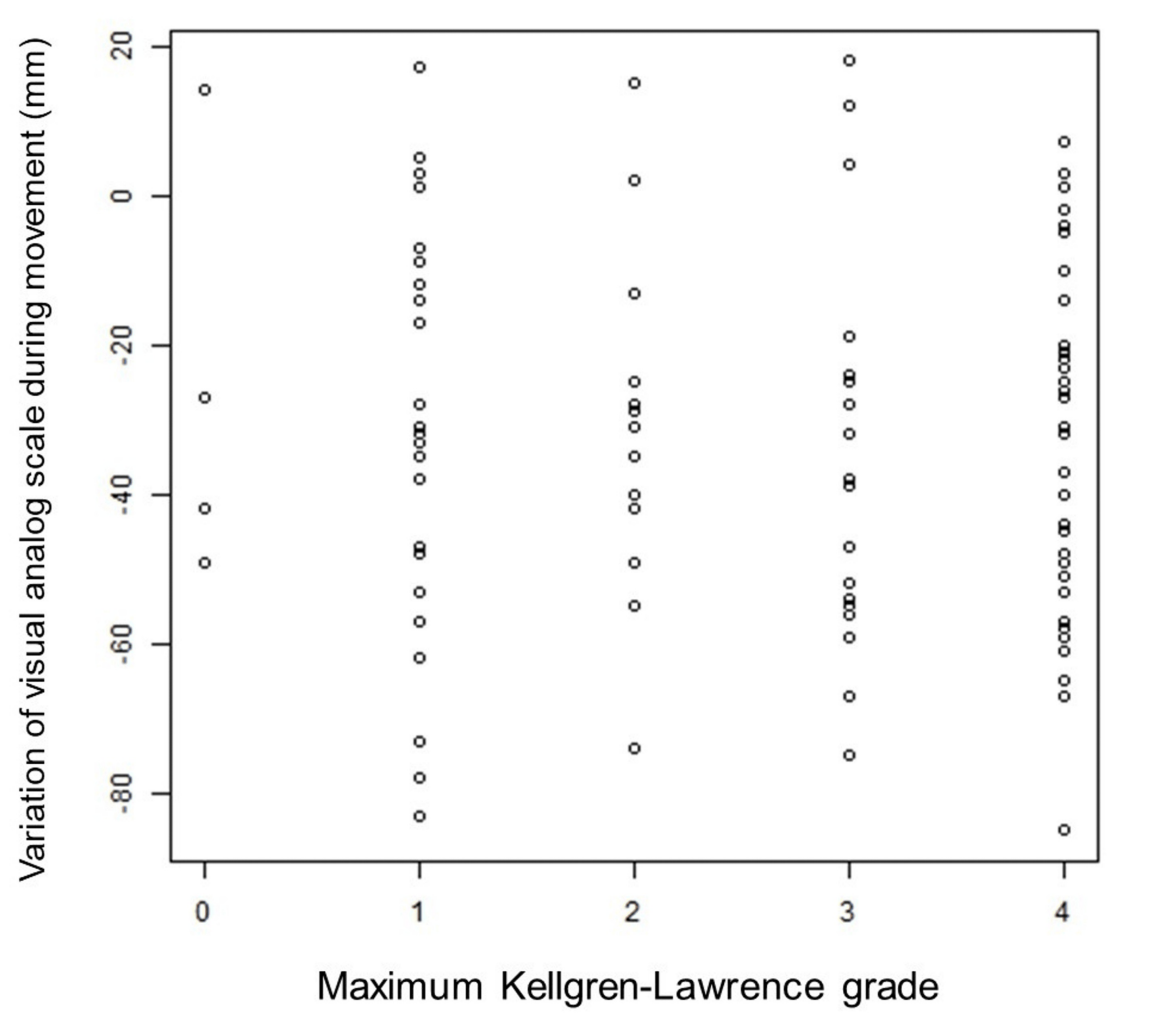
Correlation between maximum Kellgren-Lawrence grade and variation of visual analog scale on movement.

**Figure 3:**
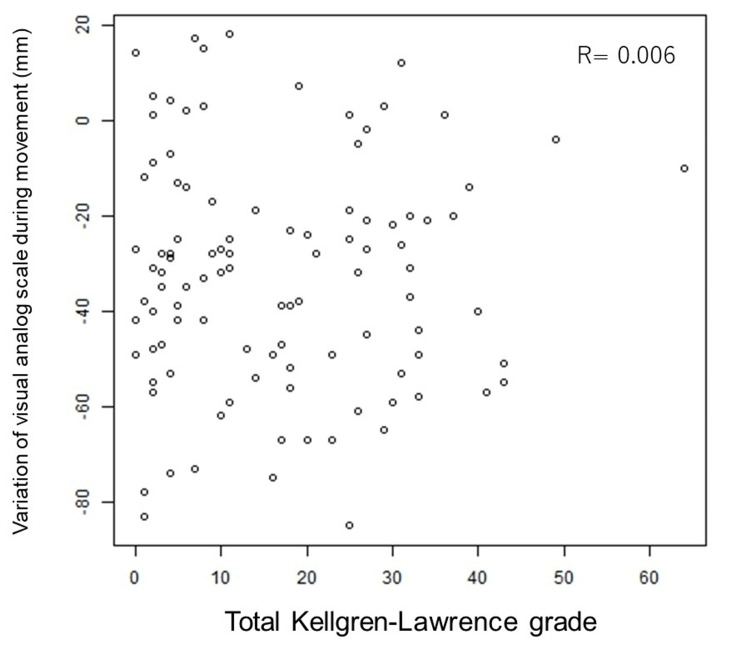
Correlation between total Kellgren-Lawrence grade (sum of KL grades for 30 joints) and variation of visual analog scale on movement.

We evaluated the correlation between the patients' maximum KL grade at baseline, the sum of KL grades of 30 joints in both hands (total KL score), treatment satisfaction, and the amount of change in VAS during movement after 12 weeks of intake of equol. In this context, the 30 joints included the thumb IP, MP, and CMC joints, as well as the DIP, PIP, and MP joints of the index to little fingers (15 joints per hand). The results showed no consistent trend by maximum KL grade (Figure 2) or total KL score (Figure 3).

**Figure 4:**
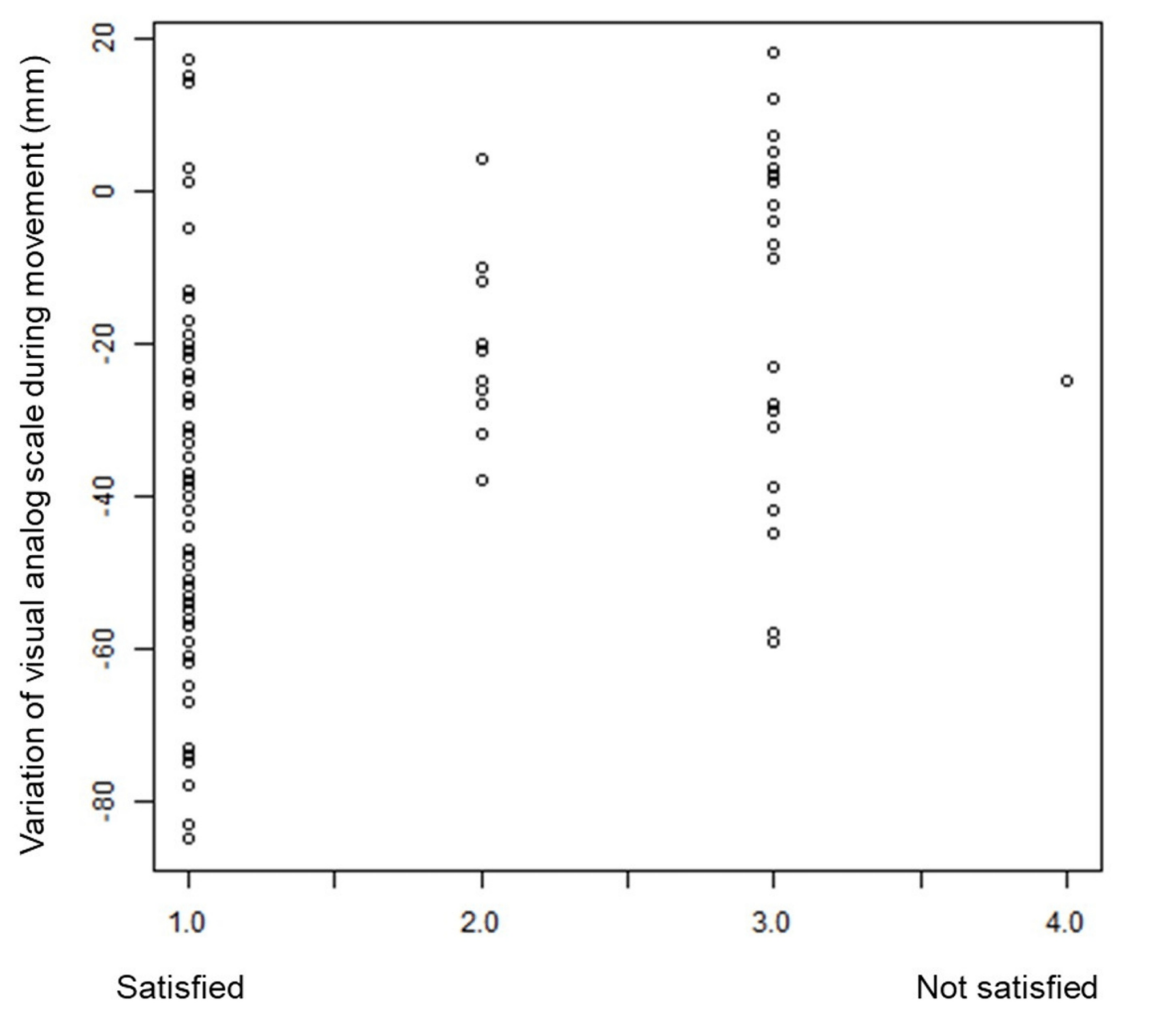
Correlation between the amount of change in VAS during movement and patient satisfaction with treatment. The scale indicates 1: satisfied with treatment, no complaints at all; 2: generally satisfied; 3: neither satisfied nor dissatisfied; 4: somewhat dissatisfied; 5: not satisfied.

After 12 weeks of treatment with equol, satisfaction with treatment was concentrated in three groups: satisfied, generally satisfied, and neither satisfied nor dissatisfied, with only one patient reporting somewhat satisfied but dissatisfied. Treatment satisfaction tended to be higher in patients with greater VAS reduction (Figure 4). No severe adverse events were observed during this study.

## Discussion

Musculoskeletal pain symptoms can appear at various ages. The frequency of such symptoms is extremely high, and they have a significant impact by decreasing the ability to function in daily life and lowering quality of life, as well as having a profound effect on life expectancy.

Osteoarthritis is a common disease that causes pain in the musculoskeletal system. Traditionally, HOA has been said to occur with overuse and aging, with a notably high rate in women in their 40s and older, although the cause is unknown. However, a large cohort study published in 2016 revealed that the prevalence of HOA in the general population in Japan is very high, almost 100% in both men and women over the age of 70, with no gender difference, and that the prevalence increases with age [19]. HOA is particularly relevant because, unlike knee or hip OA, it often remains underdiagnosed despite its high prevalence and significant impact on hand function and daily activities [19].

Treatment for patients with osteoarthritic changes on simple radiographic examination and pain in the same region has included analgesics and other drug therapies, as well as orthotics as a nonpharmacologic treatment. Although some patients respond well to medication and orthotics, the effectiveness of these treatments is generally limited, and surgical treatment is indicated when the patient's desire for pain relief is strong, taking into consideration the degree of pain, the effectiveness of conservative treatment, and the duration of the illness [1].

Hand pain in women has recently been reported to be a symptom of menopause, and a rapid decrease in estrogen may induce joint pain in the fingers. Taking equol, an estrogen-like substance, may improve hand pain in menopausal women [2]. The intake of equol may represent a new approach to pain relief for HOA in menopausal women.

A previous randomized controlled study examined the efficacy of equol on hot flashes, a symptom of menopause [6], but no reports have examined the effect of equol on hand pain in detail. Against this background, this study was designed to investigate the efficacy of equol on pain associated with HOA in menopausal women.

This is the first prospective clinical trial of equol supplement as a treatment for pain caused by HOA. The primary outcome, VAS on movement, improved with statistically significant differences after 4, 8, and 12 weeks of equol intake versus baseline. Equol intake improved the pain caused by the disease. In addition, VAS improvement was observed within a relatively short period of 1-3 months after the equol intake. DASH score and EQ-5D-5L also showed significant improvement during the test period. Function and quality of life were both suggested to be improved.

The main purpose of this study was to evaluate the improvement of pain on movement caused by HOA, but we also evaluated the improvement of various other menopausal symptoms as secondary outcomes. Significant improvements were shown in the typical menopausal symptoms of hot flashes, sweating, insomnia, and stiff neck and shoulders, which were similar to the results of a previous double-blind comparative study [6].

A limitation of this study is that it was conducted as a single-arm, open-label pilot trial without a control group. Therefore, although improvements in pain and function were observed, the improvements observed in this study should be interpreted with caution, as they may be influenced by the placebo effect or natural symptom variation. Therefore, while our results suggest the potential benefit of equol supplementation, further validation in a placebo-controlled, double-blind randomized trial is necessary to confirm its efficacy.

Although this study relied on patient-reported outcomes such as the VAS and the DASH score, these measures were selected intentionally to capture the patients' subjective experiences of pain and upper limb function. As hand osteoarthritis is a condition in which patient perception of symptoms and disability plays a central role in clinical decision-making, we believe that PROMs are appropriate and clinically meaningful endpoints. In our planned randomized controlled trial, we will continue to use these validated measures to assess the impact of equol, as they directly reflect the patients' perspective and quality of life. All patients were Japanese, and the number of patients in the cohort could be considered small. A multi-center trial would seek to confirm the results.

In addition, while the study observed a high level of treatment acceptability and minimal side effects, it was not powered to detect rare or delayed adverse events. Moreover, the absence of a control group limits the ability to evaluate the safety profile of equol in comparison to placebo or other treatments. These factors should be taken into account when interpreting the safety findings. Equol is a non-steroidal, plant-derived compound with a higher binding affinity for estrogen receptor β (ERβ) than for estrogen receptor α (ERα). Because ERβ is mainly expressed in bone, synovium, and vascular tissues, and only minimally in the endometrium and breast, equol is thought to exert limited proliferative effects on reproductive tissues. Therefore, unlike conventional estrogen replacement therapy, additional progesterone administration is not considered necessary to prevent endometrial hyperplasia. Moreover, previous large-scale epidemiological studies in Japan have reported that higher soy isoflavone intake is associated with a significantly reduced risk of breast cancer, particularly in postmenopausal women [20]. As equol is a major metabolite of daidzein, this finding supports the view that equol intake through soy consumption does not increase breast or uterine cancer risk. Nevertheless, long-term data specifically on equol remain limited, and further research is needed to confirm its safety profile.

Another limitation of this pilot study is that the KL grades for radiographic assessment were evaluated by the corresponding author alone without blinding. This single-rater, non-blinded evaluation could introduce measurement bias. However, in the ongoing randomized controlled trial based on this study, radiographic assessments are conducted by two independent orthopedic surgeons specializing in hand osteoarthritis, who are blinded to participants’ clinical data, to enhance objectivity and reproducibility.

Based on the results of this pilot study, we are planning a multicenter, placebo-controlled, double-blind, randomized, parallel group study to evaluate the effect of equol on pain improvement in perimenopausal women with HOA (HOPE-HAND Study).

## Conclusions

This pilot study suggests that equol intake may help reduce pain caused by HOA in perimenopausal women, with noticeable effects observed within a short period of 1-3 months. The significant improvement in the DASH score indicates that alleviating hand pain may be associated with better upper limb function. However, these findings should be interpreted with caution due to the study’s single-arm design, and confirmation through a randomized controlled trial is warranted.
